# Gender Disparities Are Not Binary

**DOI:** 10.1016/j.jacadv.2025.101920

**Published:** 2025-06-20

**Authors:** Stephen C. Cook, Caroline Ong, Matthew Carazo

**Affiliations:** a Franciscan Physician Network, Indiana Heart Physicians, Indianapolis, Indiana, USA; b Zucker School of Medicine at Hofstra/Northwell, Hempstead, New York, USA; c UC San Diego Health, San Diego, California, USA

We thank Fatunde et al^1^ for recognizing historic gender disparities in cardiology and the need to build an equitable workforce. Yet, the term gender is not defined in this manuscript. The term “gender” refers to socially constructed characteristics of women, men, and gender-diverse individuals.^2^ Still, many cardiologists and trainees do not recognize the entirety of this term and confuse gender with sex in clinical care, training, and cardiovascular research.^3^ In light of the myriad attacks on transgender people in the United States, it is disappointing that there is no mention of transgender or gender-diverse women throughout this State-of-the-Art Review.

As a result, this population will remain marginalized and stigmatized in cardiology despite the effort of these authors to eliminate disparities for all women. In contrast to the American College of Cardiology, the Association of Medical Colleges routinely collects sexual orientation and gender identity data (SOGI) of their enrollees. For 2024 enrollees, there were no transgender women, 0.4% genderqueer, and 0.7% nonbinary applicants.^4^ These numbers are not representative of the current U.S. population where 9.3% of U.S. adults identify as lesbian, gay, bisexual, transgender, and queer (LGBTQ+) and thus inadequate to build a resilient and equitable cardiology workforce. A recent survey study of Fellows in Training and Early Career cardiologists revealed one-third of respondents identified as LGBQ+ and 5% identified as transgender or nonbinary.^5^ Yet, Women in Cardiology have yet to publish similar data without routine SOGI data collection. The dearth of SOGI data in cardiology further perpetuates disparities for transgender and gender-diverse women.

Poor representation and visibility within cardiology create a hostile learning and working environment for transgender as well as cisgender women. Like cisgender women, many transgender women report hearing derogatory terms or are subjected to overt or covert harassment. This may lead transgender physicians to avoid disclosing of their gender identity, from participating fully within their professional body or seeking leadership positions and may dissuade them from the field of cardiology altogether. This lack of representation, along with the stressors as well as discrimination and stigma that challenge transgender men and women are associated with disparities in care and increased cardiovascular disease among LGBTQ + patients.

Although Fatunde et al provide solutions to augment mentorship, leadership and reduce harassment, we would encourage this group to broaden their scope beyond the binary (men/women) when using the term gender and gender inequities in cardiovascular medicine and offer inclusive solutions for all women.
